# Möglichkeiten und Grenzen von Ethikberatung im Rahmen der COVID-19-Pandemie (Stand: 31.03.2020): Ein Diskussionspapier der Akademie für Ethik in der Medizin

**DOI:** 10.1007/s00481-020-00580-4

**Published:** 2020-04-29

**Authors:** Akademie für Ethik in der Medizin e. V., Georg Marckmann, Gerald Neitzke, Annette Riedel, Silke Schicktanz, Jan Schildmann, Alfred Simon, Ralf Stoecker, Jochen Vollmann, Eva Winkler, Christin Zang

**Affiliations:** Humboldtallee 36, 37073 Göttingen, Deutschland

Das deutsche Gesundheitswesen steht durch die schnell steigende Anzahl an COVID-19-Erkrankten vor erheblichen Herausforderungen. In dieser Krisensituation sind alle Beteiligten mit ethischen Fragen konfrontiert, beispielsweise nach gerechten Verteilungskriterien bei begrenzten Ressourcen und dem gesundheitlichen Schutz des Personals angesichts einer bisher nicht therapierbaren Erkrankung. Daher werden schon jetzt klinische und ambulante Ethikberatungsangebote verstärkt mit Anfragen nach Unterstützung konfrontiert. Wie können Ethikberater*innen Entscheidungen in der Krankenversorgung im Rahmen der COVID-19-Pandemie unterstützen? Welche Grenzen von Ethikberatung sind zu beachten? Bislang liegen hierzu noch wenige praktische Erfahrungen vor. Angesichts der dynamischen Entwicklung erscheint es der Akademie für Ethik in der Medizin (AEM) wichtig, einen Diskurs über die angemessene Rolle der Ethikberatung bei der Bewältigung der vielfachen Herausforderungen durch die COVID-19-Pandemie zu führen und professionelle Hinweise zu geben.

Mit dem vorliegenden Diskussionspapier möchte die AEM einen Beitrag zur Beantwortung wesentlicher Fragen leisten, die sich für die Ethikberatung in den verschiedenen Bereichen des Gesundheitswesens stellen. Sie regt an, diesen Diskurs weiter zu führen und hat ein Online-Forum (s. unten) eingerichtet, in dem Ethikberater*innen ihre Erfahrungen teilen und die professionelle Selbstreflexion der Ethikberatung in Pandemiezeiten mit Anregungen fördern können.

## Wie können sich Ethikberatungsangebote auf die Pandemie-Situation einstellen?

Die Erfahrungen der letzten Wochen zeigen, dass vermehrt Anfragen an Ethikberatungsgremien herangetragen wurden. Es ist davon auszugehen, dass sich die Situationen in den Krankenhäusern und auch im außerklinischen Bereich – insbesondere in stationären Pflegeeinrichtungen – mit der zunehmenden Anzahl von COVID-19-Erkrankten weiter verschärfen wird. Es erscheint deshalb jetzt sinnvoll und notwendig, dass bei der Erstellung von örtlichen Versorgungs- und Ablaufplänen wesentliche ethische Fragen (s. oben) erkannt und beachtet werden. Ethikberater*innen können sich in diese Planungsprozesse einbringen (s. unten) sowie durch Informations- und Fortbildungsformate die Fachkenntnisse der Beteiligten vor Ort erhöhen. Dadurch können die medizinethische Kompetenz verbessert und angesichts der drängend-belastenden klinisch-ethischen Entscheidungsnotwendigkeiten allen Beteiligten Orientierungshilfe und ein Stück Sicherheit gegeben werden. Gleichzeitig kann dabei falschen Erwartungen an die Ethikberatung frühzeitig entgegentreten werden.

Weiterhin sollte frühzeitig geplant werden, wie Ethikberatungsgremien mit den vermutlich zunehmenden Anfragen an ethischer Entscheidungsunterstützung umgehen können. Dafür ist es sinnvoll zu klären, welche Kompetenzen verfügbar sind und welche zeitlichen Ressourcen die Mitglieder zur Verfügung stellen können – auch mit Blick auf die sicher weiter zunehmenden Verpflichtungen der Ethikberater*innen in der Krankenversorgung. Ggf. kann es sinnvoll sein, einen Plan auszuarbeiten, welches Mitglied zu welcher Zeit für Konsultationen zur Verfügung stehen kann. Auch die klare Benennung von Grenzen der Entscheidungsunterstützung wird in den zu erwartenden Krisensituationen hilfreich sein.

## Wie können Ethikberater*innen zur Entwicklung von fairen Verteilungs- und Priorisierungsentscheidung beitragen?

Allokationsethische Probleme im Kontext von Triage-Situationen^1^ unterscheiden sich von den klinisch-ethischen Fragestellungen, die üblicherweise Gegenstand von Ethikberatung sind. Sie sind bislang auch nicht Gegenstand des Curriculums für Ethikberatung. Nur wenige Ethikberater*innen werden deshalb über spezifische Fachkenntnisse und professionelle Erfahrung auf diesem Gebiet verfügen. Es kann daher in der Regel nicht die Aufgabe von Ethikberater*innen sein, selbst Kriterien und Verfahren für Triage-Entscheidungen zu entwickeln, zumal das grundsätzliche Vorgehen in Triage-Situation im Gesundheitswesen einheitlich erfolgen und die örtliche Umsetzung von der ärztlichen Leitung der Gesundheitseinrichtungen verantwortet werden muss. Ethikberater*innen können aber die Mitarbeitenden ihrer Einrichtung über die in einschlägigen Empfehlungen von Fachgesellschaften und anderen Institutionen benannten Kriterien informieren und bei der praktischen Umsetzung dieser Kriterien z. B. in entsprechenden Verfahrensanweisungen oder spezifischen Dokumentationsbögen für Therapieentscheidungen mitwirken. Hierzu haben inzwischen die meisten Kliniken eine „Corona-Taskforce“ eingerichtet, an denen sich Ethikberater*innen beteiligen können. Die Mitglieder von Ethikberatungsgremien sollten daher auf dem neuesten Stand sein und sich über die inhaltlichen und prozeduralen Kriterien der Triage in den Handlungsempfehlungen informieren.^2^

## Welche Formate von Ethikberatung sind in der aktuellen Situation hilfreich?

Die bei klinisch-ethischen Fragestellungen bewährten Formen der Ethik-Fallberatung z. B. in Form prospektiver oder retrospektiver ethischer Fallbesprechungen mit allen Beteiligten werden angesichts der aktuellen Situation (eingeschränkte Sozialkontakte, begrenzte Zeit- und Personalressourcen) möglicherweise nicht so zielführend sein und in der Praxis wohl nur eingeschränkt zum Einsatz kommen können. Ethikberater*innen sind deshalb gefordert, Ideen für alternative Formen von Ethik-Fallberatung zu entwickeln, die schnell abrufbar und umsetzbar sind. Ideen hierzu sind Telefon- oder Videoberatungen, Zuschaltung per Telefon bei kollegialen Beratungen über Triage-Entscheidungen oder das Training von stationären Teams, ein ethisches Time Out in Triage-Situationen durchzuführen.

## Worauf sollten Ethikberater*innen bei der Beratung von Triage-Entscheidungen achten?

Sofern Ethikberater*innen bei der Erarbeitung von Verfahrensanweisungen oder bei kollegialen Beratungen zu Triage-Entscheidungen beteiligt sind, sollten sie zunächst darauf achten, dass Triage-Kriterien bei der Zuteilung (intensiv-)medizinischer Ressourcen nur dann zum Einsatz kommen, wenn tatsächlich eine Triage-Situation vorliegt. Es wäre beispielsweise nicht gerechtfertigt, Patient*innen mit lebensbedrohlicher Erkrankung nicht zu behandeln, um Intensivkapazität für eine spätere Phase der Pandemie freizuhalten. Die Priorisierungsregeln, z. B. bei der Intensivbehandlung, gelten für alle Patient*innen unabhängig davon, ob sie an einer COVID-19-Erkrankung oder einer anderen Erkrankung leiden. Der Patientenwille bzw. der vorausverfügte Patientenwille (z. B. in Form einer Patientenverfügung) soll frühzeitig ermittelt werden. Da überwiegend ältere Menschen und Personen mit Vorerkrankungen an COVID-19 schwer erkranken, kann bei diesen Patient*innen überdurchschnittlich häufig das Vorliegen einer Vorausverfügung erwartet werden. Eine intensivmedizinische Behandlung gegen den Willen der Patient*in verstößt nicht nur gegen das ethische Prinzip der Beachtung des Patientenwillens, sondern gefährdet in Situationen knapper Behandlungsressourcen auch das Überleben anderer Patient*innen. Weiter können Ethikberater*innen darauf achten, dass keine rechtlich und ethisch problematischen Kriterien angewendet werden, wie beispielsweise eine Allokation nach dem kalendarischen Alter oder den zu gewinnenden Lebensjahren, die zu einer Altersdiskriminierung führen. Zum dritten können Ethikberater*innen durch Dokumentation und institutionsweite Einbeziehung dazu beitragen, dass Triage-Entscheidungen mit einer Konsistenz über verschiedene Behandlungs- und Versorgungseinheiten hinweg getroffen werden. Eine Konsistenz auch über regionale Versorgungsstrukturen hinweg trägt zur Gerechtigkeit bei. Hierzu können eine Vernetzung und ein Erfahrungsaustausch von Ethikberater*innen in der Region hilfreich sein.

## Was können Ethikberater*innen tun, um Mitarbeitenden bei psychischen Belastungen im Umgang mit moralischen Konflikten zu helfen

Vor dem Hintergrund der COVID-19-Pandemie ist verstärkt damit zu rechnen, dass Anfragen an klinische Ethikberater*innen gerichtet werden, bei denen der Umgang mit der psychischen Belastung von Mitarbeitenden von Krankenhäusern im Vordergrund steht. Angesichts des erhöhten, kontinuierlichen Bedarfs an medizinischer Versorgung sollte der aktive Schutz und Selbstschutz von Mitarbeitenden im Gesundheitswesen einen besonderen Stellenwert erhalten und das betrifft gleichermaßen ihre psychische wie ihre physische Gesundheit.

Bei dem Angebot einer klinischen Ethikberatung, die auf eine Verständigung über die ethisch am besten begründete Behandlungsentscheidung ausgerichtet ist, wird die psychische Belastung der Beteiligten häufig nicht explizit adressiert. Eine spezifische Beratung bei psychischen Belastungen ist auch nicht Aufgaben von Ethikberatung. Ethikberater*innen können im Rahmen einer Fallberatung dadurch zur psychischen Entlastung der Teilnehmenden beitragen, indem sie

- qualifiziert auf die Gewissensnot belasteter Mitarbeitenden eingehen („Welche widerstreitenden moralischen Werte stehen hinter der Verzweiflung/dem schlechten Bauchgefühl?“),
- Handlungsalternativen auf ihre moralische Bewertung hin prüfen („Was könnten wir noch tun?“),
- die besondere Tragik der aktuellen dilemmatischen Entscheidungssituation benennen („Bestimmte moralische Grundüberzeugungen passen aktuell nicht widerspruchsfrei zusammen.“) und
- auf bereits bestehende Strukturen, die zur Entlastung von Mitarbeitenden beitragen können, aufmerksam machen („Wer in der Klinik kann außerdem helfen?“). Darüber hinaus kann es je nach Situation vor Ort notwendig sein, dass Klinische Ethikberater*innen auf die Einrichtung von neuen Angebotsstrukturen hinwirken, um Mitarbeitende mittel- und langfristig zu unterstützen.^3^

## Wo können sich Ethikberater*innen informieren und austauschen?

Die AEM bietet aktuell wöchentliche Zoom-Konferenzen sowie ein Online-Forum (https://forum.aem-online.de) zum Austausch an. Informationen hierzu sowie über einschlägige Handlungsempfehlungen finden Sie auf der Homepage der AEM (www.aem-online.de). Gerne können Sie auch Kontakt mit der Geschäftsstelle aufnehmen (E-Mail: kontakt@aem-online.de).

## Caption Electronic Supplementary Material





## Footnotes

1 Unter einer Triage-Situation verstehen wir eine Situation, in der die vorhandenen medizinischen Ressourcen nicht ausreichen, um alle lebensbedrohlich Erkrankten zu behandeln, und eine Einteilung der Erkrankten in unterschiedlich priorisierte Kategorien erfolgt.

2 Die klinisch-ethischen Empfehlung von DIVI, DGINA, DGAI, DGIIN, DGP, DGP und AEM zu „Entscheidungen über die Zuteilung von Ressourcen in der Notfall- und Intensivmedizin im Kontext der COVID-19-Epidemie“ sowie weitere Empfehlungen und Materialien finden Sie auf der Homepage der AEM (www.aem-online.de).

3 Eine Ad-Hoc-Arbeitsgruppe in der AEM hat eine Handreichung mit dem Titel „Psychische Belastungen von Gesundheitspersonal im Umgang mit moralischen Konflikten“ im Kontext der COVID-19-Epidemie erarbeitet. Diese enthält Hinweise zu klinisch-ethischen Support-Angeboten. Die Handreichung finden Sie auf der Homepage der AEM (www.aem-online.de).

