# Supplementary material for: Möglichkeiten und Grenzen von Ethikberatung im Rahmen der COVID-19-Pandemie (Stand: 31.03.2020): Ein Diskussionspapier der Akademie für Ethik in der Medizin
Source: Ethik Med. 2020 Apr 29;32(2):195–9. [Article in German] doi: 10.1007/s00481-020-00580-4 (PMC7189629; doi:10.1007/s00481-020-00580-4)
Supplement: Supplementary file 1 [file 481_2020_580_MOESM1_ESM.docx]

**
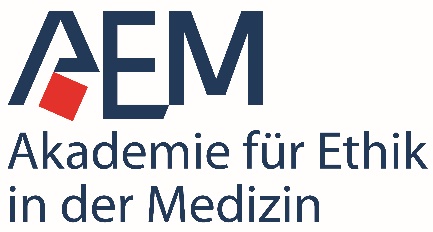
**

**Possibilities and limits of institutional ethics services in response to the COVID-19 pandemic (March 31, 2020)^[[1]](#footnote-1)^**

A discussion paper by the Academy of Ethics in Medicine^[[2]](#footnote-2)^

**Address**:

German Academy of Ethics in Medicine

Humboldtallee 36

37073 Göttingen

E-Mail: [office@aem-online.de](mailto:office@aem-online.de)

The German healthcare system is facing significant challenges due to the rapidly increasing number of COVID-19 patients. In this crisis, everyone involved in care is confronted with ethical questions, as to fair distribution criteria for limited resources and the health protection of staff in view of a so far untreatable disease. Therefore institutional and ambulatory ethics services are increasingly being confronted with requests for support. How can ethics consultants support healthcare decisions in the COVID-19 pandemic? What are the limits of ethics support service? So far there is little practical experience. In view of the dynamic development, the Academy of Ethics in Medicine (AEM) considers it important to start and entertain a discourse on the appropriate role of ethics consulting services in coping with the multiple challenges posed by the COVID-19 pandemic and to provide professional advice.

With the present discussion paper, the AEM wants to contribute to answering essential questions that arise for ethics support services in the various areas of the healthcare system. The AEM suggests continuing this discourse and has set up an online forum (see below) in which ethics consultants can share their experiences and encourage the professional self-reflection of ethics advice in pandemic times.

**How can ethics support services prepare for the pandemic situation?**

The experience of the past few weeks shows that inquiries to institutional ethics services are on the rise. Presumably, the situation in the hospitals and also outside the hospital – especially in inpatient nursing facilities – will become more serious with the increasing number of COVID-19 patients. It is therefore necessary that essential ethical questions are recognized and taken into account when planning for supply, allocation and standards of care in crisis. Ethics consultants can get involved in these planning processes (see below) and they can increase specialists’ knowledge in ethics through information and training formats on site. This can improve medical ethical competence and can provide guidance and a bit more of certainty for those confronted with the urgent and stressful clinical-ethical decision-making needs. At the same time, false expectations of ethics services can be countered if ethics consultants become involved at an early stage.

Furthermore, it should be planned early on how ethics support services can deal with the probably increasing requests for ethical decision support. It makes sense to clarify which competencies are available and which time resources the members of ethics services can make available – also with a view to the certainly increasing obligations of those ethics consultants with duties in health care. If necessary, it may be useful to develop a plan which member can be available for consultation at what time. The clear definition of the limits of decision support will also be helpful in the expected crisis situation.

**How can ethics consultants contribute to the development of fair distribution and prioritization decision?**

Ethical problems of allocation in the context of triage situations^[[3]](#footnote-3)^ differ from the clinical-ethical questions that are usually the subject of ethics consultations. So far, they are not part of the curriculum for ethics consultants. Therefore, few ethics consultants will have specific expertise and professional experience in this area. Normally, it is not a task for ethics consultants themselves to develop criteria and procedures for triage decisions, especially since the basic principles and procedures for triage should be uniformly determined for the healthcare system and their local implementation is the responsibility of the medical management of each healthcare facility.

However, ethics consultants can inform the employees of their institution about the criteria specified in the relevant recommendations of professional associations and other institutions and they can assist implementing these criteria, for example, through appropriate procedural instructions or specific documentation sheets for triage decisions. Most hospitals have now set up a corona task force in which ethics consultants can participate. The members of institutional ethics services should therefore be up to date and inform themselves about the material and procedural criteria of the triage addressed in the current recommendations of professional societies.^[[4]](#footnote-4)^

**Which formats of ethics support service are helpful in the current situation?**

The established forms of ethical case consultation that have proven of value in clinical-ethical questions, e.g. in the form of prospective or retrospective ethical case discussions with all parties involved, may not be as effective given the current situation (limited social contacts, limited time and personnel resources) and may only be of limited use in practice. Ethics consultants are therefore required to develop ideas for alternative forms of ethics case counseling that are readily available and feasible. Ideas for this include telephone or video consultations, dial in for peer advice on triage decisions, training of ICU and triage teams on an ethical time out in triage situations.

**What should ethics consultants look for when advising triage decisions?**

If ethics consultants are involved in the development of procedural instructions or in collegial counselling on triage decisions, they should first ensure that triage criteria for allocating (intensive) medical resources are only used if a triage situation is indeed on hand. For example, it would not be justified not to treat patients with life-threatening diseases in order to keep intensive care capacity free for a later phase of the pandemic. Hence, the prioritization rules, e.g. for intensive care, apply to all patients regardless of whether they suffer from COVID-19 or another illness.

Second, the patient's preferences or the patient's living will (e.g. in the form of an advance directive) should be determined at an early stage. Since predominantly older people and people with previous illnesses become seriously ill with COVID-19, an above average availability of advance directives can be expected in these patients. Intensive medical treatment against the will of the patient not only violates the ethical principle of respecting patients’ autonomy, but also jeopardizes the survival of other patients in situations of scarce treatment resources.

Third, ethics consultants can also ensure that no legally and ethically problematic triage criteria are used that lead to age discrimination, such as allocation based on chronological age or the number of years of life gained.

Fourth, through documentation and institution-wide involvement, ethics consultants can help ensure that triage decisions are made with consistency across different treatment and care units. Consistency across regional supply structures also contributes to justice. Networking and exchange of experience from ethics consultants in the region can be helpful here.

**What can ethics consultants do to help employees with psychological stress when dealing with moral conflicts?**

During the COVID-19 pandemic, requests dealing with psychological stress of hospital staff are most likely to increase and to be directed to clinical ethics consultants. In view of the increased, continuous demand for medical care, the active protection and self-protection of employees in the health care system should be given special priority, and this affects both their mental and physical health. When offering clinical ethics advice the psychological stress on those involved is often not explicitly addressed since consultation is geared towards reaching an agreement on the most ethically sound treatment decision. Specific advice on psychological stress is neither the task nor the expertise of ethics consultants. Still, in the context of ethical case consultation ethics consultants can contribute to the psychological relief of the participants by

a) responding in a qualified way to the troubled conscience of employees ("What conflicting moral values are behind the despair / bad gut feeling?",

b) assessing alternative options for action for their moral soundness ("What else could we do?"),

c) addressing the particular tragedy of the dilemma of the current decision-making situation ("Currently, certain basic moral beliefs do not fit together without contradictions.") and

d) pointing out already existing structures which can help to relieve employees, ("Who in the clinic can also help?").Depending on the situation on site, it may also be necessary for clinical ethics consultants to work towards the establishment of new offers in order to support employees in the medium and long term.^[[5]](#footnote-5)^

**Where can ethics consultants get and exchange information?**

The AEM currently offers weekly zoom conferences and an online forum (<https://forum.aem-online.de>) for exchange. Information on this as well as relevant recommendations for action can be found on the AEM homepage ([www.aem-online.de](http://www.aem-online.de)). You are also welcome to contact the office ([office@aem-online.de](mailto:office@aem-online.de)).

**Conflict of interest** The authors declare that they have no competing interests.

1. The German version „Möglichkeiten und Grenzen von Ethikberatung im Rahmen der COVID-19-Pandemie (Stand: 31.03.2020)“ is available under <https://doi.org/10.1007/s00481-020-00580-4> [↑](#footnote-ref-1)
2. As authors have contributed to this paper (in alphabetical order): Georg Marckmann, Gerald Neitzke, Annette Riedel, Silke Schicktanz, Jan Schildmann, Alfred Simon, Ralf Stoecker, Jochen Vollmann, Eva Winkler, Christin Zang. Translated by Eva Winkler. [↑](#footnote-ref-2)
3. By a triage situation, we mean a situation in which the available medical resources are not sufficient to treat all life-threatening patients and the ill are divided into differently prioritized categories. [↑](#footnote-ref-3)
4. You can find the clinical-ethical recommendations from DIVI, DGINA, DGAI, DGIIN, DGP, DGP and AEM on "Decisions on the allocation of resources in emergency and intensive care medicine in the context of the COVID-19 epidemic" as well as further recommendations and materials on the AEM homepage (www.aem-online.de).). [↑](#footnote-ref-4)
5. An ad hoc working group in the AEM has prepared a handout entitled "Psychological stress on health workers in dealing with moral conflicts" in the context of the COVID-19 epidemic. This contains information on clinical-ethical support offers. You can find the handout on the AEM home page ([www.aem-online.de](http://www.aem-online.de)). [↑](#footnote-ref-5)
